# Analysis of Adaptive Evolution in *Lyssavirus* Genomes Reveals Pervasive Diversifying Selection during Species Diversification

**DOI:** 10.3390/v6114465

**Published:** 2014-11-19

**Authors:** Carolina M. Voloch, Renata T. Capellão, Beatriz Mello, Carlos G. Schrago

**Affiliations:** Department of Genetics, Federal University of Rio de Janeiro, CEP: 21941-617, Brazil; E-Mails: carolvoloch@gmail.com (C.M.V.); renatacapellao@gmail.com (R.T.C.); biaumello@gmail.com (B.M.)

**Keywords:** rabies virus, molecular adaptation, positive selection, *Chiroptera*

## Abstract

*Lyssavirus* is a diverse genus of viruses that infect a variety of mammalian hosts, typically causing encephalitis. The evolution of this lineage, particularly the rabies virus, has been a focus of research because of the extensive occurrence of cross-species transmission, and the distinctive geographical patterns present throughout the diversification of these viruses. Although numerous studies have examined pattern-related questions concerning *Lyssavirus* evolution, analyses of the evolutionary processes acting on *Lyssavirus* diversification are scarce. To clarify the relevance of positive natural selection in *Lyssavirus* diversification, we conducted a comprehensive scan for episodic diversifying selection across all lineages and codon sites of the five coding regions in *lyssavirus* genomes. Although the genomes of these viruses are generally conserved, the glycoprotein (*G*), RNA-dependent RNA polymerase (*L*) and polymerase (*P*) genes were frequently targets of adaptive evolution during the diversification of the genus. Adaptive evolution is particularly manifest in the glycoprotein gene, which was inferred to have experienced the highest density of positively selected codon sites along branches. Substitutions in the *L* gene were found to be associated with the early diversification of phylogroups. A comparison between the number of positively selected sites inferred along the branches of RABV population branches and *Lyssavirus* intespecies branches suggested that the occurrence of positive selection was similar on the five coding regions of the genome in both groups.

## 1. Introduction

*Lyssavirus* is a genus of the *Rhabdoviridae* family (order *Mononegavirales*) comprising 11 to 15 species of single-stranded negative sense RNA viruses [1,2]. *Lyssaviruses* infect a variety of mammalian hosts, causing rabies or rabies-like pathological conditions [3,4]. The genomes of all *Lyssavirus* contain five structural genes, which are named, from 3' to 5', *N*, *P*, *M*, *G* and *L* [5,6]. The *N* gene expresses a nucleoprotein; the *P* and *M* genes encode a phosphoprotein and a matrix protein, respectively, whereas the *G* gene expresses a glycoprotein that functions in viral recognition of the host cell and therefore, it is a determining factor in the pathogenicity of infection [3,7,8]. Finally, the *L* gene expresses an RNA-dependent RNA polymerase [6,9].

The species-level classification of *Lyssavirus* has been traditionally based on the similarity of the nucleoprotein gene [2,10], and an 80%–82% sequence identity cut-off has been typically used as a standard for species delimitation [2,11]. This value, however, evidently varies when considering the whole genome. Nevertheless, *Lyssavirus* diversity has been arranged into at least three phylogroups based on serological analysis [11,12]. Phylogroup I is the major clade of the genus, comprising nine species, including the rabies virus RABV. Phylogroup II, the second most diverse clade, comprises three species with strictly African distribution [3,13]. This phylogroup contains the Mokola virus (MOKV), which infects mammals in sub-Saharan Africa and is one of the few *Lyssavirus* species that has not been isolated from bats [3]. The genetically divergent West Caucasian bat virus (WCBV) is the only species of Phylogroup III [11]. This virus was initially isolated in southeastern Europe, but its geographic range has been posteriorly expanded after the identification of WCBV from *Miniopterus* bats in Kenya [14]. Apart from these three phylogroups, the recent isolation of the Ikoma virus (IKOV) from an African civet with clinical rabies in the Serengeti National Park of Tanzania has greatly expanded *Lyssavirus* genetic diversity [15,16]. The IKOV, which was not identified in bats, has not been assigned to any of the existing phylogroups [12].

Phylogroup I is of particular epidemiological interest because of widespread human rabies. According to the World Health Organization, RABV is responsible for approximately 60,000 deaths per year, and the majority of cases have been reported in Asia and Africa. The genetic diversity of phylogroup I presents a marked geographical pattern [17]. RABV fundamentally comprises two major lineages: the first lineage circulates in terrestrial mammals with worldwide distribution, and the other lineage has been exclusively identified in the New World [17,18,19]. The history of RABV has been thoroughly investigated, particularly in North America, where several cross-species transmissions have been reported [20].

Interest in the evolution of *Lyssavirus* species has prompted researchers to investigate their evolutionary history, associated with mammalian hosts or reservoir switching, the timescale of diversification of the group and the biogeography of* Lyssavirus* [8,21,22,23]. Therefore, evolutionary analyses of this genus have typically focused on pattern-based methods and have not examined the effects of evolutionary forces during species diversification. However, after analyzing the glycoprotein and nucleocapsid genes of RABV, Holmes* et al.* [24] reported the occurrence of positive selection acting upon codon sites of the coding sequences, whereas Szanto* et al.* [25] inferred positively selected codon sites on the *L* gene.

Because of its complex evolutionary history of host switching, the diversification of *Lyssavirus* is an appealing case study to investigate episodes of diversifying selection along lineages. The analysis of diversifying evolution, however, has been hindered by the large computational burden of the majority of the existing methods. The development of maximum likelihood methods that identify lineage-specific evolution [26] and the posterior extension of these methods to accommodate branches and codon sites under positive selection was a major step in the study of adaptive evolution [27]. To save computational time, however, these methods require *a priori* specification of the branches in which the *d*_N_/*d*_S_ ratio can be greater than one. This consists of a significant limitation, as it is biologically difficult to assign which lineage experienced positive selection and, hence, statistical issues associated with multiple testing typically arise [28]. Recently, Pond* et al.* [29] derived a computational method that scans for diversifying selection across the branches of a phylogeny without *a priori* assignment of the lineages, thereby facilitating the analysis of adaptive evolution. Moreover, Murrell* et al.* [30] later proposed a mixed effects model of evolution (MEME) to infer specific codon sites that underwent positive selection along branches.

In the present study, we performed a global analysis of diversifying selection during the evolution of all *Lyssavirus* species. Using complete genomes, we scanned all five genomic coding sequences for episodes of adaptive evolution. We observed that positive selection played a significant role in the early diversification of the phylogroups. The polymerase *L* gene was the primary target of episodic diversifying selection during early diversification of the *Lyssavirus* phylogroups, whereas the *G* gene was coding region with the highest density of positively selected sites along lyssavirus diversification. Our results also showed that IKOV and WCBV might comprise a monophyletic group, validating the inclusion of these viruses in phylogroup III. In addition to the polymerase, several codon sites were inferred to have undergone adaptive evolution on the glycoprotein. Because a similar amount of positively selected codon sites was found within RABV and between *Lyssavirus* species, we propose that the macroevolutionary dynamics of lyssaviruses are long-term consequences of intraspecies dynamics.

## 2. Materials and Methods

### 2.1. Sequences and Alignment

All complete genomes available for *Lyssavirus* species were downloaded from public databases, and two datasets were assembled in order to test for the consistency of the results to taxonomic sampling. Taxon sampling is relevant in our analysis because the number of *Lyssavirus* genomes available is greatly unbalanced, favoring RABV genomes. This may result in topological shape with spurious statistical values [31]. Firstly, a dataset containing up to three randomly sampled genomes for each representative species was composed. To compose the second dataset we eliminated clades comprising genetic distances <0.04 in order to obtain the maximum sample size that is computationally feasible. The resulting sets contained 50 and 75 genomes, respectively, and will be hereafter named *small* and *large* datasets (Table S1). Sequence alignments for each gene were independently conducted using the MAFFT program [32]. Nucleotide sequences were aligned based on the translated amino acid sequences to maintain the reading frame, as required for diversifying selection analyses. To identify the location of the root of the *Lyssavirus* tree, an additional data set containing the full sample of representative genomes available for *Rhabdovirida*e species was also assembled.

### 2.2. Evolutionary Analysis

Each gene alignment was subjected to an analysis of intragenic recombination. Recombination screening was performed using the single break point method available on the Datamonkey server (www.datamonkey.org). We observed no intragenic recombination on the five coding sequences. Phylogenetic inference was conducted using Phyml 3 [33] and MrBayes 3.2 [34] under the evolutionary model selected according to the likelihood ratio test (LRT) available in the HyPhy package [35]. Model rejection was set at *p*-value < 0.05. We also scanned for recombination using a supermatrix composed by concatenating the five coding genes. Because no recombination was estimated, tree topologies inferred from the supermatrix was used throughout the analysis.

The analysis of diversifying selection along the branches and codon sites was independently implemented for the five coding regions. The random effects branch-site (BSR) and the MEME methods implemented in the Datamonkey server were used. MEME analysis was conducted using an evolutionary model based on the LRT algorithm available in Datamonkey. In MEME, codon-specific positive selection was admitted at *p*-value < 0.05, and the inference of the lineages in which diversifying selection occurred at a given codon was performed using a Bayes empirical Bayes approach [30]. Branches with sites in which the Bayes factor was greater than 1 were considered as targets of episodic diversifying selection. The BSR analysis was performed using the MG94 model, adopting three categories for the *d*_N_/*d*_S_ ratio. In the BSR method, rejection of the null model was made at corrected *p*-value < 0.05 [29].

The comparison of the density of codon sites under episodic diversifying selection within the RABV population branches and along the branches associated with the interspecies *Lyssavirus* diversity, we have also used MEME estimates. To perform such comparison, we have counted the occurrence of positively selected codon sites along lineages within each category (RABV population-level branches* vs.* interspecies branches) for the five genomic coding regions. We then conducted the Fisher’s exact test to verify whether the distribution of the amount of positively selected codons sites along branches in the five genomic regions differed between the two branch categories.

## 3. Results

**Figure 1:**
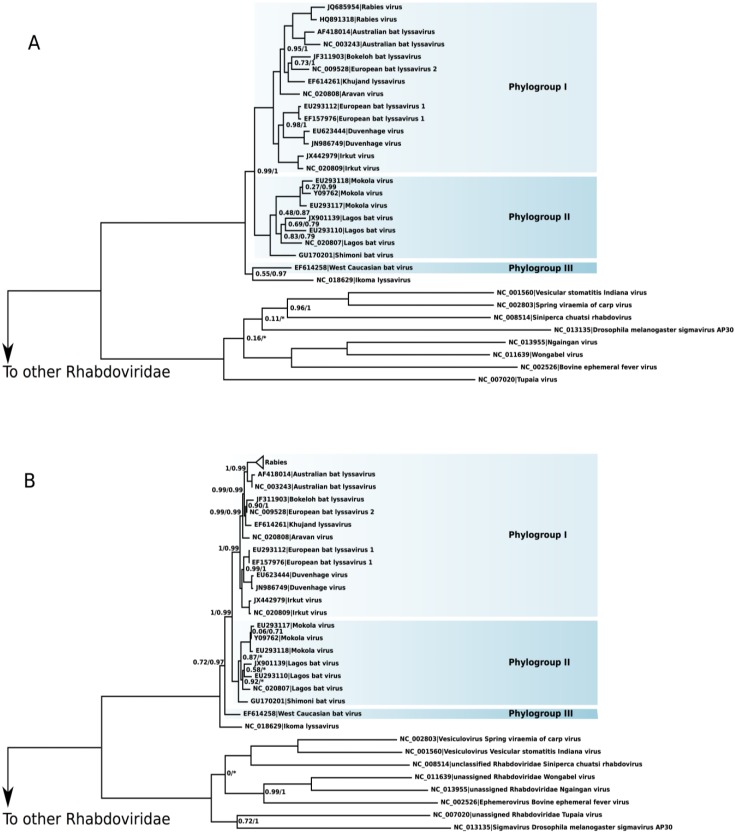
Rooted phylogenies of *Lyssavirus*. The numbers on nodes indicate the aLRT statistics/Bayesian posterior probability. Nodes without numbers are fully supported: 1.0/1.0. (**A**) Phylogeny of *Lyssavirus* using the small data set and (**B**) large data set.

The phylogeny of *Lyssavirus* recovered the species as monophyletic (Figure 1). Both small and large datasets presented the same major phylogenetic relationships. The exception was the evolutionary relationship of the IKOV and the WCBV. In the small data set, the IKOV was the sister species of WCBV (aLRT 55%, BPP 97%), whereas in the large data set the IKOV was the sister group of the remaining *Lyssavirus* (aLRT 72%, BPP 97%). To test the significance of these alternative phylogenetic associations of the IKOV, we have conducted the Kishino-Hasegawa topological test [36]. In the small data set, the null hypothesis that considered the log-likelihoods of both topological associations as equal was rejected in favor of the (IKOV + WCBV) phylogeny (*p* < 0.01). In the large data set, however, the null hypothesis was not rejected (*p* = 0.27) and, thus, the log-likelihood of the (IKOV + WCBV) phylogeny is statistically equivalent to the topology depicted in Figure 1B. The Australian bat virus was confirmed as the sister species of RABV in both trees.

**Figure 2:**
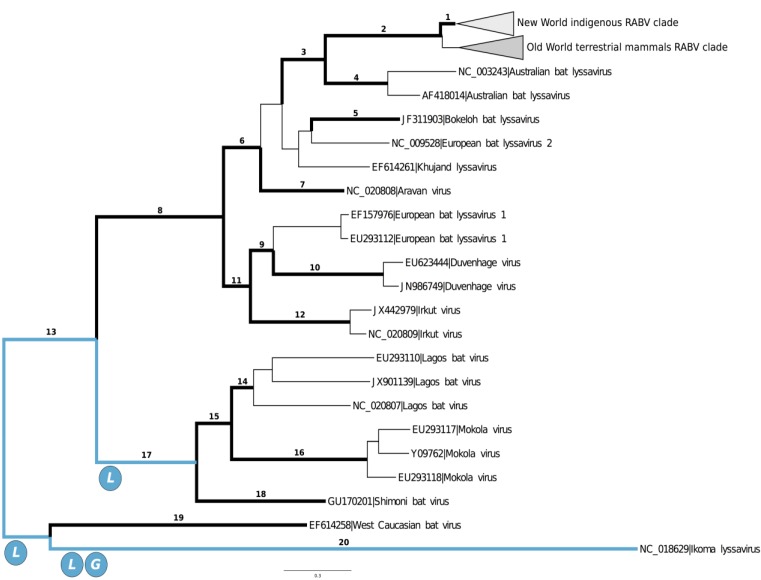
Unrooted phylogeny of *Lyssavirus* displaying the results of MEME and BSR analyses. Branches colored blue are those lineages inferred by BSR to have undergone episodic diversifying selection on the genes shown within circles. Thick branches indicate those in which the MEME inferred diversifying selection, numbers in branches relate to the detailed description of codon sites at the ω > 1 class using the Bayes empirical Bayes approach.

The results of the analysis of diversifying selection was consistent in both datasets, thus results are presented for the large dataset solely. The inference of diversifying selection along the branches conducted by the BSR algorithm indicated that the *L* and *G* genes underwent adaptive molecular evolution (Figure 2). The events of adaptive evolution were primarily located along the basal branches of the *Lyssavirus* phylogeny. In the *L* gene, the BSR test identified four branches that tested positive for episodic diversifying selection (corrected *p*-value < 0.05). At the branch leading to one of the IKOV sequences (NC_018629), the proportion of codon sites evolving with ω > 1 was inferred at 13%, with ω = 14.4. Approximately 4% of the sites were inferred to have undergone adaptive evolution along the branch that separates the (IKOV + WCBV) group from the remaining *lyssavirus*. At the basal branch of the clade grouping SHIBV, MOKV and LBV, 3% of the sites also presented ω > 1. Moreover, 1% of the sites of the *L* gene were inferred to have undergone adaptive evolution in one of the external branches of RABV. In the *G* gene, the BSR method inferred diversifying selection in only one branch, the branch leading to the IKOV sequence (NC_018629), in which an estimated 10% of the codon sites evolved with ω > 1.

The MEME analysis presented a scenario of pervasive adaptive evolution at codon sites during the diversification of *Lyssavirus* species (Figure 2). All five coding regions presented at least one codon site under ω > 1 in one of the branches, and the codons inferred under the ω^+^ class were recurrently detected along the same branches (Table 1). The number of codon sites along lineages assigned to ω > 1 was smaller in the *M* and *N* genes. In the *M* gene, codon site 31 was inferred with ω > 1 at the basal branch of IKOV, whereas in the *N* gene, codon site 101 was inferred under episodic diversifying evolution at the branch leading to the MOKV + LBV and the branch leading to the Bokeloh bat virus. The *P* gene presented only two codon sites with ω^+^ during the diversification of lyssaviruses, and site 117 was estimated to have undergone adaptive evolution at the basal branch of the IKOV. However, codon 157 of the *P* gene showed the most episodes of diversifying selection in the *Lyssavirus* genome, and this site was assigned to the ω^+^ class at five branches, the majority of which were identified during the basal diversification of *lyssavirus* species.

**Table 1:** Codons inferred to be under episodic diversifying selection by the BEB approach at the branches displayed in Figure 2.

Branch	Gene	Codon	Count of Synonymous Substitutions: Count of Non-Synonymous Substitutions
1	L	1967	0.5:1.5
2	L	223	0.5:1.5
	G	226	0.83:2.17
3	G	530	0.5:1.5
	G	226	0:1
4	G	113	0.67:2.33
5	N	101	0:1
6	L	205	0:1
7	G	18	0.5:2.5
8	L	1820	0:1
	G	530	0.33:2.67
	G	353	1.67:2.83
9	G	530	0:1
10	P	157	1:1
	L	223	0:1
	L	1967	0:1
	G	113	1:1
11	L	423	0.5:2.5
12	G	276	0.5:2.5
13	P	117	0:3
	P	157	0.5:2.5
	L	1967	0:1
	L	290	0:1
	G	530	0:2
	G	113	0:1
	G	18	0:1
14	L	2098	0:1
15	N	101	0:1
16	G	2	0:2
17	L	223	0.5:2.5
	L	1968	0:1
	G	18	0:1
18	P	157	0.5:2.5
19	P	157	0:2
	L	1812	0:1
20	M	31	0.5:1.5
	P	157	0:1
	L	223	0.5:2.5
	L	1820	0:1
	L	1968	1:1

Consistent with the results of the BSR analysis, a large number of codon sites with ω > 1 were identified in the *G* and *L* genes. In the *G* gene, codons 18, 113 and 530 were inferred at the ω^+^ class in more than two branches. All these branches comprised internal branches leading to major lineages. For example, codon site 530 underwent consecutive episodes of adaptive evolution, initially during the diversification of the IKOV + WCBV clade and the remaining lyssaviruses and also during the separation between phylogroup II and phylogroup I. The *L* gene was the coding region with the most abundant number of codons under the ω^+^ class (9), and two sites, 223 and 1967, repeatedly underwent episodic selection during *Lyssavirus* evolution. The same pattern was observed in the other genes, showing that diversifying selection occurred at internal branches, indicating species-level diversification events.

Notably, the diversification of RABV from other *lyssavirus* species involved episodic selection on the *G* and *L* genes. We estimated that codon 226 of the *G* gene and codon 223 of the *L* gene underwent diversifying selection along the branch separating RABV from ABLV. We also inferred that codon 1967 of the *L* gene underwent adaptive evolution during the diversification of the two major RABV groups (Figure 2, Table 1).

The comparison between the number of the positively selected codon sites within the RABV and those inferred at the branches associated with the diversification of the *Lyssavirus* species showed similar population-level and interspecies selective patterns (Table 2). The occurrence of positively selected codon sites within RABV exceeds the number of sites in the ω^+^ class found in interspecies branches; however, a contingency test comparing the interspecies and population-level number of positively selected codon sites on branches along the five genes failed to reject the null hypothesis of equal distribution (*p*-value = 0.26),

**Table 2:** Codon sites undergoing positive selection within RABV species, as inferred by the BSR method using the Bayes Empirical Bayes approach.

Gene	RABV Population
N	436, 114, 101, 36
P	30, 117, 157
M	31, 158
G	18, 70, 113, 114, 115, 117, 125, 128, 226, 276, 353
L	205, 223, 315, 316, 370, 389, 423, 660, 683, 728, 856, 1319, 1427, 1493, 1569, 1640, 1643, 1798, 1812, 1813, 1820, 1822, 1823, 1836, 1967, 1968, 2090, 2098

## 4. Discussion

The occurrence of episodic diversifying selection at the basal lineages of the *Lyssavirus* demonstrates that adaptive molecular evolution has played a role in several diversification events within the genus. Taken together, the results of the BSR and MEME analyses suggested that the genomes of lyssaviruses have undergone episodes of molecular adaptation. The BSR analysis also showed that the *L* gene was the main target of adaptive evolution during the early diversification of phylogroups. The product of the* L* gene interacts with several host cell factors that affect the efficiency of viral propagation, making this region an immediate target of molecular adaptation to different host environments [19]. The *G* gene, encoding the surface glycoprotein, was the second most genomic region in which episodes of adaptive evolution were inferred on the basal *lyssavirus* branches by BSR.

The MEME analysis along the entire topology estimated a greater amount of positively selected codon sites on the *L* and *G* genes, respectively. If the probability of undergoing diversifying evolution is homogeneous along the genome, a higher occurrence of positively selected codon sites is expected because the *L* and *G* genes are the larger coding regions. Overall, when the frequency of positively selected sites was measured per codon site, the *G* gene was the genomic region inferred as the primary target of diversifying selection during *lyssavirus* diversification, followed by the *P* and *L* genes. When MEME estimates were restricted to the basal branches identified by BSR analysis (branches 20, 17 and 13 in Figure 2), the *P* gene was the genomic region with the highest density of positively selected sites, followed by the *G* and *L* genes. However, the evaluation of MEME results from those three basal lineages only should be interpreted with caution, because of the high rates of false positives. Moreover, the BSR algorithm failed to identify the *P* gene as undergoing episodic diversifying selection on the early *lyssavirus* splits. Although we consider the overall comparison of the MEME results to be robust, the analysis of a restricted number of branches may be biased.

Previous surveys of positively selected sites along *Lyssavirus* genomes have been focused on within species diversity only, particularly for RABV. Therefore, a comparison of the estimates obtained herein with the results of these studies might not be meaningful. This factor is evident for the *L* gene, in which intraspecies analyses have rarely detected positively selected codon sites. However, Szanto* et al.* [25] inferred codon site 62 is a target of adaptive evolution in this coding region. Although the *L* gene was inferred as the main target of diversifying selection on the early diversification of phylogroups by BSR, we have not observed codon site 62, or any other nearby site, under positive selection on the same branched by MEME analysis.

In the *N* gene, which is highly conserved among lyssaviruses. Consistent with the analysis of Bourhy* et al.* [37], we inferred that codon site 101 underwent diversifying selection. Hughes* et al.* [38] also analyzed the *N* gene and failed to find evidence of adaptive evolution on this gene. Interestingly, we observed that the same coding region was under positive selection within both RABV and other *Lyssavirus* species. Regarding the *G* gene, Holmes* et al.* [24] identified codon sites 189 and 370 to have undergone adaptive evolution. Moreover, site 189 is highly variable and might impact cell tropism, as this region is adjacent to antigenic site II within a neurotoxin-like region that might be responsible for binding to the nicotinic acetylcholine receptor, a putative RABV receptor. It is intriguing thus that codon position 189 of the *G* gene was not inferred under episodic diversifying selection.

Interestingly, we identified positively selected substitutions in the *P*, *M* and *L* genes and also inferred diversifying selection through BSR analysis of the branch leading to IKOV. Compared RABV, IKOV is the most genetically divergent *lyssavirus* species, and this species is also antigenically distinct, poorly responding to current rabies vaccine strains [16].

It has been suggested that IKOV and WCBV might comprise a monophyletic grouping [16]. However, it is not possible to evaluate this claim using unrooted tree topologies. Few studies have inferred the phylogeny of *Lyssavirus* through rooting the clade using other members of *Rhabdoviridae*. Bourhy* et al.* [39] conducted an analysis using the *L* gene, although the results of this study did not resolve the evolutionary relationships of the lineage. Delmas* et al.* [5] rooted the *Lyssavirus* tree using the midpoint, but this analysis did not include IKOV and WCBV. Tao* et al.* [21] also conducted an analysis of *Lyssavirus* evolution using the Bayesian method implemented in BEAST software. In the present study, we showed that IKOV and WCBV might comprise a monophyletic clade, because the K-H test failed to reject the (IKOV + WCBV) topology in the larger data set. As the genetic distance between both viruses is large, we suggest that the diversity of this lineage is likely underestimated, and the application of modern sequencing methods will possibly increase the diversity of *Lyssavirus* species antigenically different from RABV. More importantly, although the IKOV + WCBV clade is genetically divergent from phylogroups I and II, these viruses induce encephalitis characteristics similar to RABV.

The identification of individual branches in which adaptation acted on a given codon site is statistically difficult [30], thus it is relevant to cross-validate the estimates using other methods. Notably, the results from the BSR and MEME analyses were consistent. The branches identified using the BSR method showed the largest number of episodes of diversifying selection in MEME. This observation was evident for the branch separating IKOV and WCBV from the remaining *lyssavirus* species (branch 13, Figure 2 and Table 1), in which the MEME algorithm inferred seven episodes of molecular adaptation along the genome.

Moreover, it is tempting to associate episodic diversifying selection with host switching in *Lyssavirus*. However, the genomes from non-RABV lyssaviruses needed to investigate this question thoroughly are scarcely available. If the original *lyssavirus* reservoir is indeed in the order *Chiroptera* [20], then it is intriguing that IKOV, the most genetically divergent lineage compared with RABV, was identified in the civet, a *Carnivora*, but IKOV was not identified in bats [16]. Nevertheless, the occurrence of WCBV in bats and the recently discovered Lleida virus [1], which are both genetically divergent from phylogroups I and II, strongly suggests that *Chiroptera* is the original *Lyssavirus* reservoir. In any case, it is reasonable to conclude that cross-species spillovers have been characteristic of *Lyssaviruses* since early diversification. Moreover, because some codon sites were subjected to intraspecific positive selection in RABV and among *Lyssavirus* species, particularly in the *N* gene, it is conceivable that the macroevolutionary dynamics acting on this genus is a long-term extension of the microevolutionary processes occurring within species. The occurrence of a greater amount of codon sites that have undergone adaptive molecular evolution in RABV populations when compared to interspecies *Lyssavirus* branches may be artifact caused by false positives, because population-level diversity commonly presents short branch lengths [40].

Finally, it is worth mentioning that the analysis of molecular adaptation along genomes should be ideally conducted on sequences that were directly obtained by sequencing lyssaviruses from the host cells, without virus replication on tissue cultures. This may artificially select codon substitutions and bias the evolutionary analysis. In fact, several genomes used in this study were obtained by sequencing virus populations from tissue cultures. To evaluate the impact of artificial selection, we have compared genomes from the same species, obtained from natural and artificial environments, to check if the genomes from tissue culture viruses accumulated an anomalous number of substitutions. In every comparison, we have not found any significant difference. For example, Irkut virus genomes used in this study were obtained using direct (JX442979) and tissue culture (NC_020809) procedures, and lengths of the branches leading to both terminals were equivalent (Figure 2). Moreover, because the aim of our analysis was the diversification of *Lyssavirus* species, and we focused on interspecies branches, artificial selection was probably not an issue.

In conclusion, the present study showed that the genomes of *Lyssavirus* underwent several events of episodic diversifying selection during the diversification of phylogroups and species. The *L* and *G* genes were the main targets of positive selection during the early diversification of phylogroups and on the lineage leading to IKOV. Overall, the *G* gene was the coding region with the highest density of adaptive evolutionary substitutions along *lyssavirus* evolution. However, we have shown that codon substitutions confined to a few sites could be associated with species-level diversification events, particularly on the basal-most branches of the *lyssavirus* phylogeny. A comparison of the number of codon sites under positive selection along the five coding regions within RABV branches presented a similar count than that observed in interspecies branches, suggesting that the mode of evolution of *Lyssavirus* genomes is homogeneous.

## Supplementary Files

Supplementary File 1
