# Supplementary material for: Analysis of Adaptive Evolution in Lyssavirus Genomes Reveals Pervasive Diversifying Selection during Species Diversification
Source: Viruses. 2014 Nov 19;6(11):4465–78. doi: 10.3390/v6114465 (PMC4246234; doi:10.3390/v6114465)
Supplement: Supplementary File 1 [file viruses-06-04465-s001.pdf]

Supplementary Materials

Analysis of Adaptive Evolution in *Lyssavirus* Genomes Reveals Pervasive Diversifying Selection during Species Diversification

Carolina M. Voloch, Renata T. Capellão, Beatriz Mello and Carlos G. Schrago

Table S1. GenBank accession numbers of sequences used in this study.

Accession	Species	Genera
EF614259	Aravan virus	Lyssavirus
NC 020808	Aravan virus	Lyssavirus
AF081020	Australian bat lyssavirus	Lyssavirus
NC 003243	Australian bat lyssavirus	Lyssavirus
AF418014	Australian bat lyssavirus	Lyssavirus
JF311903	Bokeloh bat lyssavirus	Lyssavirus
NC 020810	Duvenhage virus	Lyssavirus
JN986749	Duvenhage virus	Lyssavirus
EU623444	Duvenhage virus	Lyssavirus
EU293120	Duvenhage virus	Lyssavirus
EU293119	Duvenhage virus	Lyssavirus
EU626551	European bat lyssavirus 1	Lyssavirus
NC 009527	European bat lyssavirus 1	Lyssavirus
EF157976	European bat lyssavirus 1	Lyssavirus
EU626552	European bat lyssavirus 1	Lyssavirus
EU293112	European bat lyssavirus 1	Lyssavirus
EU293109	European bat lyssavirus 1	Lyssavirus
EU293114	European bat lyssavirus 2	Lyssavirus
EF157977	European bat lyssavirus 2	Lyssavirus
NC 009528	European bat lyssavirus 2	Lyssavirus
JX193798	Ikoma lyssavirus	Lyssavirus
NC 018629	Ikoma lyssavirus	Lyssavirus
JX442979	Irkut virus	Lyssavirus
EF614260	Irkut virus	Lyssavirus
NC 020809	Irkut virus	Lyssavirus
EF614261	Khujand lyssavirus	Lyssavirus
EU293108	Lagos bat virus	Lyssavirus
NC 020807	Lagos bat virus	Lyssavirus
EU293110	Lagos bat virus	Lyssavirus
JX901139	Lagos bat virus	Lyssavirus
EU259198	Lagos bat virus	Lyssavirus
GU170202	Lagos bat virus	Lyssavirus
FJ905105	Lyssavirus Ozernoe	Lyssavirus
EU293117	Mokola virus	Lyssavirus
EU293118	Mokola virus	Lyssavirus
NC 006429	Mokola virus	Lyssavirus
Y09762	Mokola virus	Lyssavirus
GU170201	Shimoni bat virus	Lyssavirus
EF614258	West Caucasian bat virus	Lyssavirus

Table S1. *Cont.*

Accession	Species	Genera
AY705373	Rabies virus	Lyssavirus
EF206713	Rabies virus	Lyssavirus
DQ099525	Rabies virus	Lyssavirus
DQ099524	Rabies virus	Lyssavirus
JQ685927	Rabies virus	Lyssavirus
JQ944708	Rabies virus	Lyssavirus
JQ685971	Rabies virus	Lyssavirus
JQ685943	Rabies virus	Lyssavirus
FJ712195	Rabies virus	Lyssavirus
EF206714	Rabies virus	Lyssavirus
EU549783	Rabies virus	Lyssavirus
AB009663	Rabies virus	Lyssavirus
EF564174	Rabies virus	Lyssavirus
EF206717	Rabies virus	Lyssavirus
EU293111	Rabies virus	Lyssavirus
EU182346	Rabies virus	Lyssavirus
EU886634	Rabies virus	Lyssavirus
EF206708	Rabies virus	Lyssavirus
DQ875051	Rabies virus	Lyssavirus
EU293121	Rabies virus	Lyssavirus
AB128149	Rabies virus	Lyssavirus
EF206710	Rabies virus	Lyssavirus
EU311738	Rabies virus	Lyssavirus
EU877067	Rabies virus	Lyssavirus
EF206709	Rabies virus	Lyssavirus
EF206719	Rabies virus	Lyssavirus
KC171643	Rabies virus	Lyssavirus
KC171645	Rabies virus	Lyssavirus
JQ647510	Rabies virus	Lyssavirus
KC171644	Rabies virus	Lyssavirus
JQ685939	Rabies virus	Lyssavirus
JQ685904	Rabies virus	Lyssavirus
JQ685906	Rabies virus	Lyssavirus
JQ685967	Rabies virus	Lyssavirus
JQ685917	Rabies virus	Lyssavirus
JQ685910	Rabies virus	Lyssavirus
JQ685919	Rabies virus	Lyssavirus
JQ685950	Rabies virus	Lyssavirus
JQ685922	Rabies virus	Lyssavirus
JQ685949	Rabies virus	Lyssavirus
JQ685954	Rabies virus	Lyssavirus
JQ685977	Rabies virus	Lyssavirus
JQ685940	Rabies virus	Lyssavirus
JQ685957	Rabies virus	Lyssavirus
JQ685896	Rabies virus	Lyssavirus
JQ685960	Rabies virus	Lyssavirus

Table S1. *Cont.*

Accession	Species	Genera
AB608731	Rabies virus	Lyssavirus
JQ685933	Rabies virus	Lyssavirus
JQ685893	Rabies virus	Lyssavirus
JQ685945	Rabies virus	Lyssavirus
JQ685944	Rabies virus	Lyssavirus
JQ685972	Rabies virus	Lyssavirus
JQ685975	Rabies virus	Lyssavirus
JQ685901	Rabies virus	Lyssavirus
EU182347	Rabies virus	Lyssavirus
AB362483	Rabies virus	Lyssavirus
HM535790	Rabies virus	Lyssavirus
AB569299	Rabies virus	Lyssavirus
HQ317918	Rabies virus	Lyssavirus
AB519642	Rabies virus	Lyssavirus
AB519641	Rabies virus	Lyssavirus
AB635373	Rabies virus	Lyssavirus
AB517660	Rabies virus	Lyssavirus
AB517659	Rabies virus	Lyssavirus
JN234411	Rabies virus	Lyssavirus
JQ685947	Rabies virus	Lyssavirus
JQ423952	Rabies virus	Lyssavirus
JQ685938	Rabies virus	Lyssavirus
JQ685941	Rabies virus	Lyssavirus
JQ685961	Rabies virus	Lyssavirus
JQ685924	Rabies virus	Lyssavirus
JQ944705	Rabies virus	Lyssavirus
JQ685937	Rabies virus	Lyssavirus
JQ685934	Rabies virus	Lyssavirus
JQ685966	Rabies virus	Lyssavirus
JQ730682	Rabies virus	Lyssavirus
AB645847	Rabies virus	Lyssavirus
JQ685968	Rabies virus	Lyssavirus
JQ685903	Rabies virus	Lyssavirus
JQ685942	Rabies virus	Lyssavirus
JQ685928	Rabies virus	Lyssavirus
JQ685905	Rabies virus	Lyssavirus
JQ685948	Rabies virus	Lyssavirus
JQ685902	Rabies virus	Lyssavirus
JQ685920	Rabies virus	Lyssavirus
JQ685895	Rabies virus	Lyssavirus
JQ685931	Rabies virus	Lyssavirus
JQ685898	Rabies virus	Lyssavirus
JQ685965	Rabies virus	Lyssavirus
JQ685953	Rabies virus	Lyssavirus
JQ944704	Rabies virus	Lyssavirus

Table S1. *Cont.*

Accession	Species	Genera
JQ685918	Rabies virus	Lyssavirus
JQ685925	Rabies virus	Lyssavirus
JQ685907	Rabies virus	Lyssavirus
JQ944706	Rabies virus	Lyssavirus
HQ891318	Rabies virus	Lyssavirus
HQ450385	Rabies virus	Lyssavirus
HQ450386	Rabies virus	Lyssavirus
KC196743	Rabies virus	Lyssavirus
JN609295	Rabies virus	Lyssavirus
FJ577895	Rabies virus	Lyssavirus
JQ946087	Rabies virus	Lyssavirus
KC595280	Rabies virus	Lyssavirus
KC595281	Rabies virus	Lyssavirus
KC595282	Rabies virus	Lyssavirus
KC595283	Rabies virus	Lyssavirus
EF542830	Rabies virus	Lyssavirus
AF499686	Rabies virus	Lyssavirus
M31046	Rabies virus	Lyssavirus
NC 001542	Rabies virus	Lyssavirus
FJ913470	Rabies virus	Lyssavirus
M13215	Rabies virus	Lyssavirus
FJ712193	Rabies virus	Lyssavirus
EU886632	Rabies virus	Lyssavirus
JQ685974	Rabies virus	Lyssavirus
JQ685915	Rabies virus	Lyssavirus
JQ685909	Rabies virus	Lyssavirus
JQ685912	Rabies virus	Lyssavirus
JQ685900	Rabies virus	Lyssavirus
JQ685894	Rabies virus	Lyssavirus
JQ685952	Rabies virus	Lyssavirus
JQ685930	Rabies virus	Lyssavirus
JQ685946	Rabies virus	Lyssavirus
JQ685935	Rabies virus	Lyssavirus
JQ944707	Rabies virus	Lyssavirus
JQ685951	Rabies virus	Lyssavirus
JQ685916	Rabies virus	Lyssavirus
JQ685970	Rabies virus	Lyssavirus
JQ685964	Rabies virus	Lyssavirus
JQ685955	Rabies virus	Lyssavirus
JQ685899	Rabies virus	Lyssavirus
JQ685936	Rabies virus	Lyssavirus
JQ685932	Rabies virus	Lyssavirus
JQ685921	Rabies virus	Lyssavirus
JQ685958	Rabies virus	Lyssavirus
JQ685976	Rabies virus	Lyssavirus
JQ685929	Rabies virus	Lyssavirus

Table S1. *Cont.*

Accession	Species	Genera
JQ685897	Rabies virus	Lyssavirus
JQ685911	Rabies virus	Lyssavirus
JQ685923	Rabies virus	Lyssavirus
JQ685973	Rabies virus	Lyssavirus
JQ685926	Rabies virus	Lyssavirus
JQ685914	Rabies virus	Lyssavirus
JQ685962	Rabies virus	Lyssavirus
JQ685956	Rabies virus	Lyssavirus
JQ685963	Rabies virus	Lyssavirus
JQ685913	Rabies virus	Lyssavirus
JQ685959	Rabies virus	Lyssavirus
JQ944709	Rabies virus	Lyssavirus
JQ685908	Rabies virus	Lyssavirus
JQ685892	Rabies virus	Lyssavirus
JQ685969	Rabies virus	Lyssavirus
JX276550	Rabies virus	Lyssavirus
GU358653	Rabies virus	Lyssavirus
JX088694	Rabies virus	Lyssavirus
JN786877	Rabies virus	Lyssavirus
JN786878	Rabies virus	Lyssavirus
AB699220	Rabies virus	Lyssavirus
GU345747	Rabies virus	Lyssavirus
JX473840	Rabies virus	Lyssavirus
GU345746	Rabies virus	Lyssavirus
JX473839	Rabies virus	Lyssavirus
GU345748	Rabies virus	Lyssavirus
JX473838	Rabies virus	Lyssavirus
JX473841	Rabies virus	Lyssavirus
KC193267	Rabies virus	Lyssavirus
AB781935	Rabies virus	Lyssavirus
EF206712	Rabies virus	Lyssavirus
EU293116	Rabies virus	Lyssavirus
EF206716	Rabies virus	Lyssavirus
EF206718	Rabies virus	Lyssavirus
DQ875050	Rabies virus	Lyssavirus
EF206711	Rabies virus	Lyssavirus
FJ866836	Rabies virus	Lyssavirus
AB085828	Rabies virus	Lyssavirus
EU886633	Rabies virus	Lyssavirus
FJ712196	Rabies virus	Lyssavirus
EF437215	Rabies virus	Lyssavirus
FJ959397	Rabies virus	Lyssavirus
GU647092	Rabies virus	Lyssavirus
GQ918139	Rabies virus	Lyssavirus
AY956319	Rabies virus	Lyssavirus
EU877071	Rabies virus	Lyssavirus

Table S1. *Cont.*

Accession	Species	Genera
EU877070	Rabies virus	Lyssavirus
EU886636	Rabies virus	Lyssavirus
EU877069	Rabies virus	Lyssavirus
GU565704	Rabies virus	Lyssavirus
EU293113	Rabies virus	Lyssavirus
EU293115	Rabies virus	Lyssavirus
EU643590	Rabies virus	Lyssavirus
FJ866835	Rabies virus	Lyssavirus
FJ712194	Rabies virus	Lyssavirus
EF206720	Rabies virus	Lyssavirus
EF206715	Rabies virus	Lyssavirus
KC169986	Rabies virus	Lyssavirus
EU886631	Rabies virus	Lyssavirus
EU877068	Rabies virus	Lyssavirus
AB044824	Rabies virus	Lyssavirus
EU886635	Rabies virus	Lyssavirus
GU565703	Rabies virus	Lyssavirus
EF206707	Rabies virus	Lyssavirus
NC 007642	Lettuce necrotic yellows virus	Cytorhabdovirus
NC 011532	Lettuce yellow mottle virus	Cytorhabdovirus
NC 002251	Northern cereal mosaic virus	Cytorhabdovirus
NC 002526	Bovine ephemeral fever virus	Ephemerovirus
NC 005093	Hirame rhabdovirus	Novirhabdovirus
NC 001652	Infectious hematopoietic necrosis virus	Novirhabdovirus
NC 000903	Snakehead virus	Novirhabdovirus
NC 000855	Viral hemorrhagic septicemia virus Fil3	Novirhabdovirus
NC 005974	Maize fine streak virus	Nucleorhabdovirus
NC 011542	Maize Iranian mosaic virus (MIMV)	Nucleorhabdovirus
NC 005975	Maize mosaic virus	Nucleorhabdovirus
NC 016136	Potato yellow dwarf virus	Nucleorhabdovirus
NC 003746	Rice yellow stunt virus	Nucleorhabdovirus
NC 001615	Sonchus yellow net virus	Nucleorhabdovirus
NC 006942	Taro vein chlorosis virus	Nucleorhabdovirus
NC 013135	Drosophila melanogaster sigmavirus AP30	Sigmavirus
NC 013955	Ngaingan virus	unassigned Rhabdoviridae
NC 007020	Tupaia virus	unassigned Rhabdoviridae
NC 011639	Wongabel virus	unassigned Rhabdoviridae
NC 008514	Siniperca chuatsi rhabdovirus	unclassified Rhabdoviridae
NC 002803	Spring viraemia of carp virus	Vesiculovirus
NC 001560	Vesicular stomatitis Indiana virus	Vesiculovirus
